# The effectiveness of mobile application for monitoring diabetes mellitus and hypertension in the adult and elderly population: systematic review and meta-analysis

**DOI:** 10.1186/s12913-023-09879-6

**Published:** 2023-08-12

**Authors:** Emily de Souza Ferreira, Fernanda de Aguiar Franco, Marina Marilac dos Santos Lara, André Amaral Levcovitz, Mateus Araújo Dias, Tiago Ricardo Moreira, Ary Henrique Morais de Oliveira, Rosângela Minardi Mitre Cotta

**Affiliations:** 1 Department of Nutrition and Health, Federal University of Viçosa, Viçosa, Brazil; 2 Department of Medicine and Nursing, Federal University Viçosa, Viçosa, Brazil; 3 Department of Computing, Federal University of Tocantins, Palmas, Brazil

**Keywords:** Mobile application, Digital health, Hypertension, Diabetes mellitus, Adult, Aged, Systematic review

## Abstract

**Context:**

Arterial Hypertension (AH) and Diabetes Mellitus (DM) are diseases that are getting worse all over the world. Linked to this advance, is the growing digital health market with numerous mobile health applications, which aim to help patients and professionals in the proper management of chronic diseases. The aim of this study was to analyze, through a systematic review and meta-analysis, the effectiveness of using mobile health applications in monitoring AH and/or DM in the adult and elderly population.

**Methods:**

The systematic review and meta-analysis was carried out in accordance with the Preferred Reporting Items for Systematic Reviews and Metanalyses guidelines and involved searching five databases – Medline/PubMed, Embase, CINAHL, Virtual Library in Health and Cochrane Library. The review included randomized and cohort clinical trials testing the effects of the intervention on changing biochemical parameters and clinical efficacy in people treated for AH and/or DM. The quality of the selected studies was assessed based on the evaluation criteria of the Joanna Briggs Institute tool. The random effects meta-analysis method was used to explain effect distribution between studies, by Stata® software (version 11.0) and publication bias was examined by visual inspection of graphs and Egger test.

**Results:**

We included 26 studies in the systematic review and 17 in the meta-analysis. These studies were published between 2014 to 2022 in 14 countries. Were reported improvement in knowledge and self-management of AH and DM, social motivation with treatment and behavioral change, reduction in glycated hemoglobin values, fasting glucose and blood pressure, improvement in adherence to drug treatment, among others. The result of the meta-analysis showed that there is evidence that the use of mobile applications can help reduce glycated hemoglobin by 0.39% compared to the usual care group.

**Conclusions:**

Monitoring and self-monitoring of behaviors and health care related to AH and DM in adults and the elderly through mobile applications, has clinically significant effectiveness in reducing glycated hemoglobin levels. Future studies should provide more evidence and recommendations for best practices and development of digital health interventions.

**Trial registration:**

PROSPERO. International Prospective Registry of Systematic Reviews. CRD42022361928.

**Supplementary Information:**

The online version contains supplementary material available at 10.1186/s12913-023-09879-6.

## Introduction

Arterial Hypertension (AH), and its aggravating factors, is responsible for 8.5 million deaths from stroke, kidney disease and ischemic heart disease worldwide [1, 2]. Currently, there are approximately 1.13 billion people in the world affected by this disease, and many of these people were unaware of the diagnosis [2]. Diabetes Mellitus (DM), another serious and growing disease, presented in 2019, an estimated that 463 million people were living with this disease, representing 9.3% of the global adult population (20 to 79 years old) [3, 4]. Estimates from the 10th edition of the IDF Diabetes Atlas is that there is a projected 16% increase in the expected prevalence of DM due to the aging population [5].

Fortunately, both AH and DM can be detected in Primary Health Care units in the communities themselves and, with proper monitoring and management and changes in behavior, it is possible to reduce risk factors that are modifiable to prevent these diseases and to avoid their complications in the long term [1, 2]. Some recent studies, including systematic reviews and meta-analyses, have concluded that there is evidence and a positive effect on the use of digital interventions in clinical practice for monitoring and following up individuals with DM and AH and their risk factors, in addition to helping healthcare professionals in the treatment of these patients [6–9]. However, more rigorous methods are still needed to explore the effects on behavioral changes, service delivery and user engagement with apps [8, 10].

With the worsening of the COVID-19 pandemic, the scenario of non-communicable chronic diseases, in particular AH and DM, became even worse, since service to the general public was discontinued, as all care and attention was focused on affected patients by COVID-19 [1]. In parallel with the increase in chronic diseases in the elderly population, the digital market, more specifically the digital mobile health (mHealth) market, also grown. Today, there are numerous mobile health applications with the main objective of helping patients monitor their own health, adhere to treatment and thus control chronic diseases, their aggravating conditions and achieve improvements in clinical conditions [1, 8–11].

Mobile applications, facilitated through digital technologies such as computers, smartphones, tablets and other mobile devices, are accessible to large numbers of people and in different settings, complementing medical services and positively affecting long-term health conditions [8, 11]. Still, there is no systematic review with meta-analysis available in the literature whose objective is to analyze the effectiveness of the use of mobile health applications for the management, monitoring and self-monitoring of adults and elderly people with AH and/or DM.

The focus of many global, regional, and national initiatives and programs is to improve effective treatment coverage of patients. With this, our hypothesis is that through a systematic review and meta-analysis, we will be able to analyze the effectiveness of mobile applications in health as a digital intervention, to improve the management, self-monitoring and monitoring of adults and elderly people with AH and/or DM and that these applications may be more effective than currently used strategies.

## Methods

### Study design and search strategy

This systematic review and meta-analysis were conducted in accordance with the Preferred Reporting Items for Systematic Reviews and Meta-Analyses (PRISMA) reporting guidelines [12] and registered with the International Prospective Register of Systematic Reviews (PROSPERO), under protocol number CRD42022361928.

The search strategy was developed using keywords from relevant literature, including previous reviews. With these terms in hand, we analyzed which ones were adequate among those established by MeSH (Medical Subject Headings) terms.

According to MeSH, the following terms were added: “mobile applications”, “diabetes mellitus”, “hypertension”, “adult”, “aged”. To take into account the digital health perspective, search terms were used in different combinations: 1) (mobile applications OR applications OR mobile health OR mobile phone) AND (diabetes mellitus OR diabetes) AND adult; 2) (mobile applications OR applications OR mobile health OR mobile phone) AND (diabetes mellitus OR diabetes) AND aged; 3) (mobile applications OR applications OR mobile health OR mobile phone) AND (hypertension OR arterial hypertension) AND adult; 4) (mobile applications OR applications OR mobile health OR mobile phone) AND (hypertension OR arterial hypertension) AND aged.

### Data sources

This systematic review involved searching the Excerpta Medica dataBASE (EMBASE), Virtual Health Library (VHL), Cumulative Index to Nursing and Allied Health Literature (Cinahls), Cochrane Library and Medical Literature Analysis and Retrieval System Online (MEDLINE/PubMed) electronic databases, in July 2022 to identify eligible studies.

### Study selection

The review included randomized and cohort clinical trials testing the effects of the intervention on changing biochemical parameters and clinical efficacy in people treated for AH and/or DM. Were considered for inclusion, trials involving adults and the elderly published after the year 2000 and without restriction on location or language. The date restriction is due to the fact that communication technologies used prior to this date no longer represent the current reality of telemedicine [13, 14] and because the Consort-eHealth statement for reporting of eHealth and mHealth interventions was released after 2010 [15].

Screening of title, abstract and full text was performed independently by three reviewers (FAF, MMSL, AAL), and disagreements were discussed by another reviewer (ESF). Each of the three reviewers selected studies for possible inclusion based on title and abstract content. Studies that filled out the inclusion criteria were analyzed in the full-text review.

Articles had to meet all following criteria to be eligible for full-text screening: (1) population comprised adults and elderly individuals treated for AH and/or DM; (2) intervention consisted of monitoring health behaviors related to AH and/or DM through a mobile application; (3) intervention was aimed at supporting changes in blood pressure, blood glucose or fasting blood glucose and related health behaviors; (4) the comparator was usual care, enhanced usual care or minimal behavioral intervention; (5) the study included measurements of blood pressure, fasting glucose, or HbA1c; and (6) the study design was a randomized controlled trial or cohort.

Exclusion criteria included: (1) lack of thematic focus (e.g., “health”, “digital”, or “mobile application” were not the main topic or were discussed only in passing); (2) studies focusing on digitalization as a means of transforming the customer experience; and (3) unpublished literature, conference and congress abstracts, commentaries, case reports, pilot studies, abstracts, reviews, letters or editorials.

### Data extraction

Data were extracted using the Rayyan – Intelligent Systematic Review application. Three authors (FAF, MMSL, AAL) extracted all data and one reviewer (ESF) analyzed data for accuracy. The following data were collected: (1) title, authors, country, study duration, publication date; kind of study; (2) number of individuals included, data collection instrument, type of mobile application used (intervention); (3) disease(s) studied, level of health care, characteristics of the individuals included, main results of the intervention.

The following were extracted from the results: (1) treatment time; (2) sample number of the control and intervention groups; (3) HbA1c values in both groups (when available in both study groups); (4) systolic and diastolic blood pressure measurements in both groups (when available in both study groups). These outcome data were extracted for baseline and endpoints in the control and intervention groups for most studies, otherwise, only endpoints were extracted. When follow-up values were missing (e.g., SD), final intervention values were selected to estimate its effects.

We created a Microsoft Excel spreadsheet to extract all the information mentioned above.

### Quality and risk of bias assessment

The quality of the selected studies was assessed based on the evaluation criteria of the Joanna Briggs Institute tool [16] specific for cohort studies and randomized controlled trials. The results were measured in percentage, attributing to each checklist item, as follows: 1 point for “YES”, 0.5 point for “not clear” and 0 for “NO”. Good quality studies were those that scored above 75% [17].

Meta-analysis was performed using fixed and randomized models (where necessary) and weighted mean difference (WMD) to calculate the intervention effects of using the mobile app on improving clinical parameters across studies. We chose to use the final values after the intervention to compare the effect of the mobile application, since the baseline results were similar between the intervention and control groups.

We calculated the standard deviation of some studies using data imputation. Heterogeneity was assessed by the chi-square test (χ2) with a significance of 90% (p < 0.10), and its magnitude was determined by the I-square (I2) [18]. Thus, heterogeneity was classified as low, moderate or high when I2 values were above 25, 50 and 75%, respectively. Heterogeneity was explored using subgroup analyzes to investigate whether study-level variables could explain the observed heterogeneity, if necessary.

The results were synthesized through meta-analysis of the mean HbA1c in the sample of the control and post-test intervention groups, with the respective standard deviation. Pressure values for both groups were not obtained, due to their absence in most articles. The choice of post-test means occurred because the groups were well balanced (i.e., similar means) at the beginning of the studies (pre-test).

Meta-analysis was performed using Stata® software (version 11.0) and publication bias was examined by visual inspection of funnel plots and the Egger test. The risk of bias was also analyzed using RevMan (version 5.4). Statistical significance of the overall effect size of mobile health app use was determined by the 95% confidence interval (CI).

## Results

### Studies identified and included

**Fig. 1:**
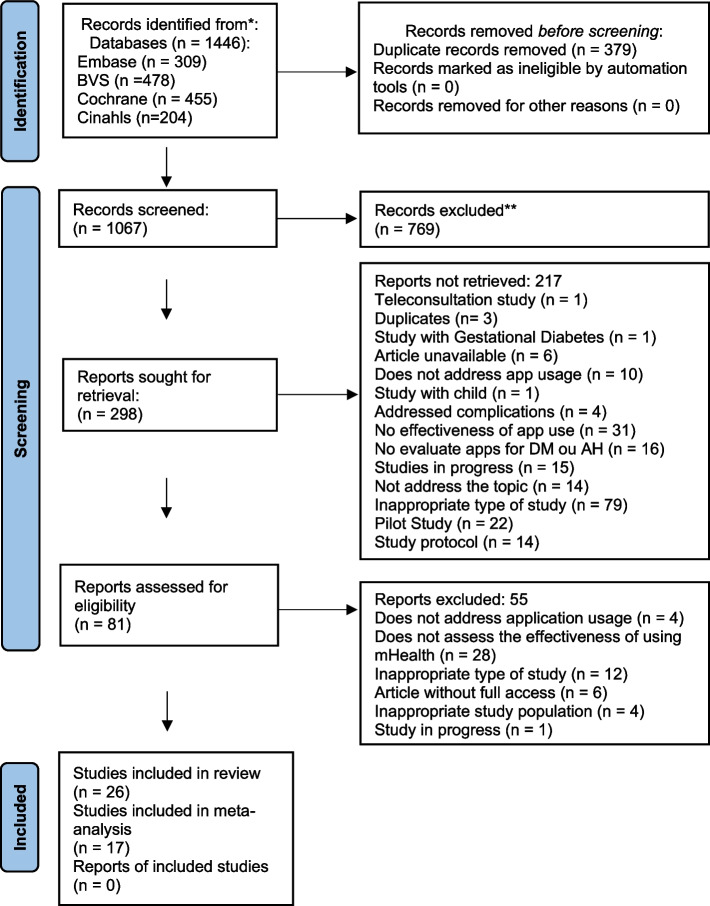
Flowchart of preferred report items for systematic reviews and meta-analyses (PRISMA) of study selection

We searched five databases and obtained 1,446 articles. The first step was to delete duplicate articles using the Rayyan app, remaining 1,067 articles. In the second stage, studies were excluded by screening titles and abstracts, remaining 81 articles. In the third stage, the studies were excluded through careful screening of the full text of the articles. Finally, we included 26 articles for the systematic review, of which 17 contained all the necessary data for inclusion in the Meta-analyses. The retrieval and selection process are shown in Fig. 1.

### Characteristics of the included studies

There were 5,288 participants in the 26 studies, from 14 countries (South Korea [*n* = 3], Singapore [*n* = 2], India [*n* = 2], Norway, Japan, China [*n* = 5], USA [*n* = 4], Sri Lanka, Spain, Indonesia, Canada, United Kingdom, Germany, Australia) and the Gaza Strip. 14 studies worked only with DM, 4 studies addressed only AH and 8 studies covered both mentioned chronic diseases.

As for the results, were addressed improvement in knowledge and self-management of DM, social motivation with treatment and behavioral change (11). The reduction of HbA1c values and fasting glycemia (15), blood pressure (2) and improvement in adherence to drug treatment and adequate diet (6), reduction in body weight (3) and adherence/improvement in physical activity (2). 7 different studies showed no improvement in the parameters analyzed, which were: blood pressure, HbA1c, and adherence to drug treatment. All these results are detailed in Additional file 1.

It is worth noting that all the studies used the mobile application in a complementary way to the methods traditionally employed in health services, i.e., they were not tested separately, as this would compromise the ethical aspects of the studies.

### Meta-analysis

Of the 26 articles included in the systematic review, 17 compared the effectiveness of using mobile applications in reducing HbA1c values.

Of the total number of studies that addressed isolated AH (4 studies) or associated with DM (8 studies), only 4 provided sufficient information to perform the meta-analysis. Therefore, this analysis was not performed for AH, only for DM.

A total of 1,478 patients were included. Of these, 723 were in the control group and 755 in the intervention group. Treatment time ranged from 2 to 12 months, with a mean of 5 months (± 2.50).

### Blood pressure

 In our systematic review, we found 4 articles that only address AH and 8 articles that address AH associated with DM. Of the 4 specific studies for AH, there were a total of 834 patients, 415 in the intervention group and 419 in the control group. Of the total of 12 studies, only half presented blood pressure values at the beginning of the study and at the end of the study, both for the intervention group and for the control group.

The mean value of systolic blood pressure in the control group at the beginning and end of the study was, respectively, 136.9 mmHg and 135.6 mmHg, while in the intervention group it was, respectively, 137.5 mmHg and 134.2 mmHg.

Most studies showed that both groups (control and intervention) showed a significant improvement in levels of adherence, with greater improvements in the intervention group in the total score. We emphasize significant results for the following categories: use of hypertensive medication; improved adherence to medication and diet. Some studies did not show significant differences between the control and intervention groups.

### Glycated hemoglobin

**Fig. 2:**
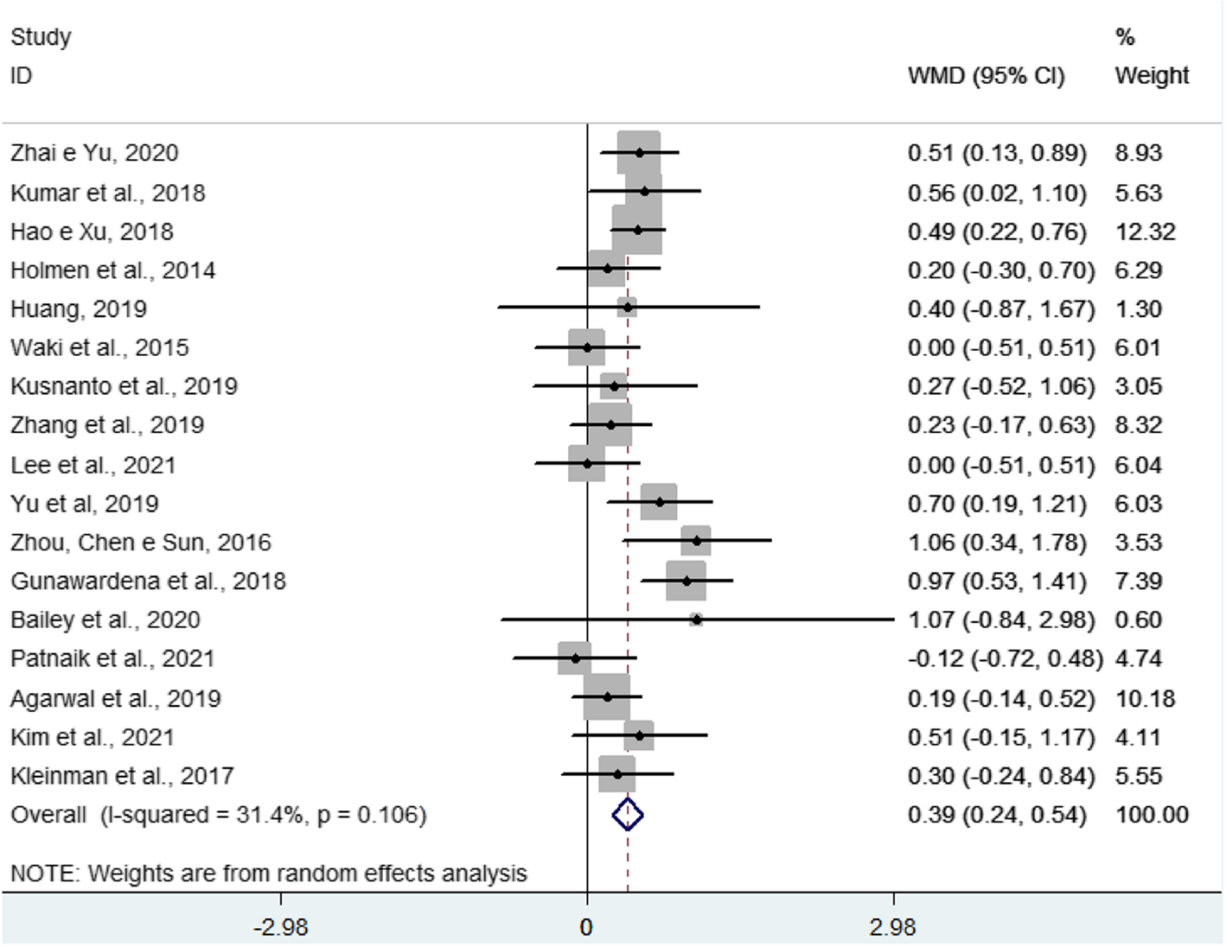
Forest plot of the effects of mobile health apps on reducing HbA1c

As shown in Fig. 2, eligible articles have a weighted mean value of reduction or increase in HbA1c and a confidence interval and weight that varies according to the sample size of the article. This figure shows that most studies indicate that the intervention group was significantly better than the control group in terms of the outcome, which would be a drop in HbA1c levels.

As a main result, we found a difference between the intervention and control groups, indicating that the intervention group, that is, those who used mobile health applications to monitor chronic disease, had a reduction of 0.39% (CI 0.24—0.54) in HbA1c value (%) compared to the usual care group (Fig. 2).

There was low heterogeneity between studies (31.4%; *p* = 0.106), which indicates little variation in study results. For this reason, subgroup and meta-regression analyzes were not performed.

### Risk of bias

**Fig. 3:**
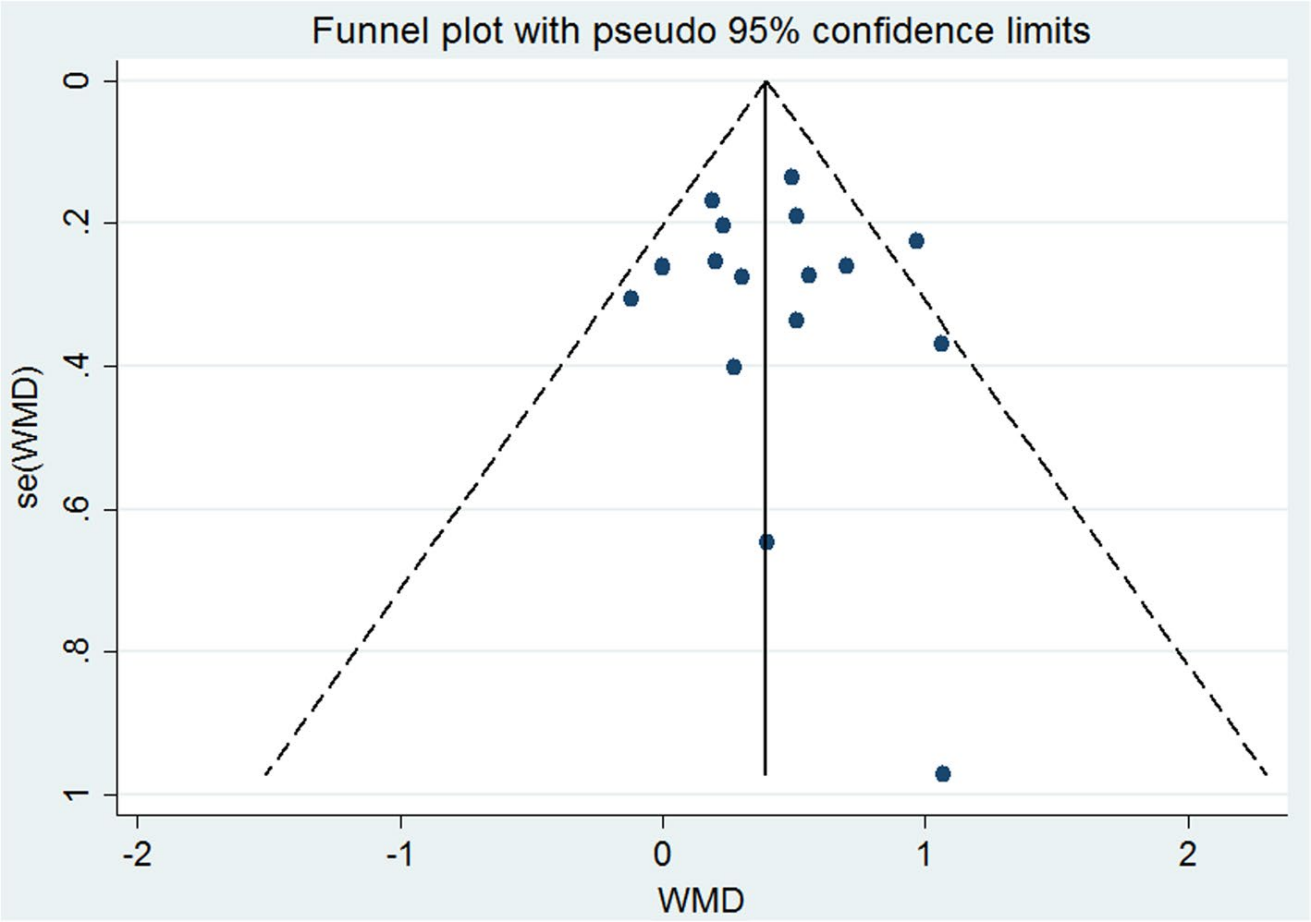
Funnel Chart. Weighted Mean Differences, Funnel Plot with 95% Confidence Limits

The Fig. 3 shows the results of the “meta funnel”, a test performed to investigate the risk of publication bias. In those analysis, there is no indicative of risk of bias. Egger's test also indicated non-significant results (*p* = 0.835), reinforcing the results obtained by the test.

**Fig. 4:**
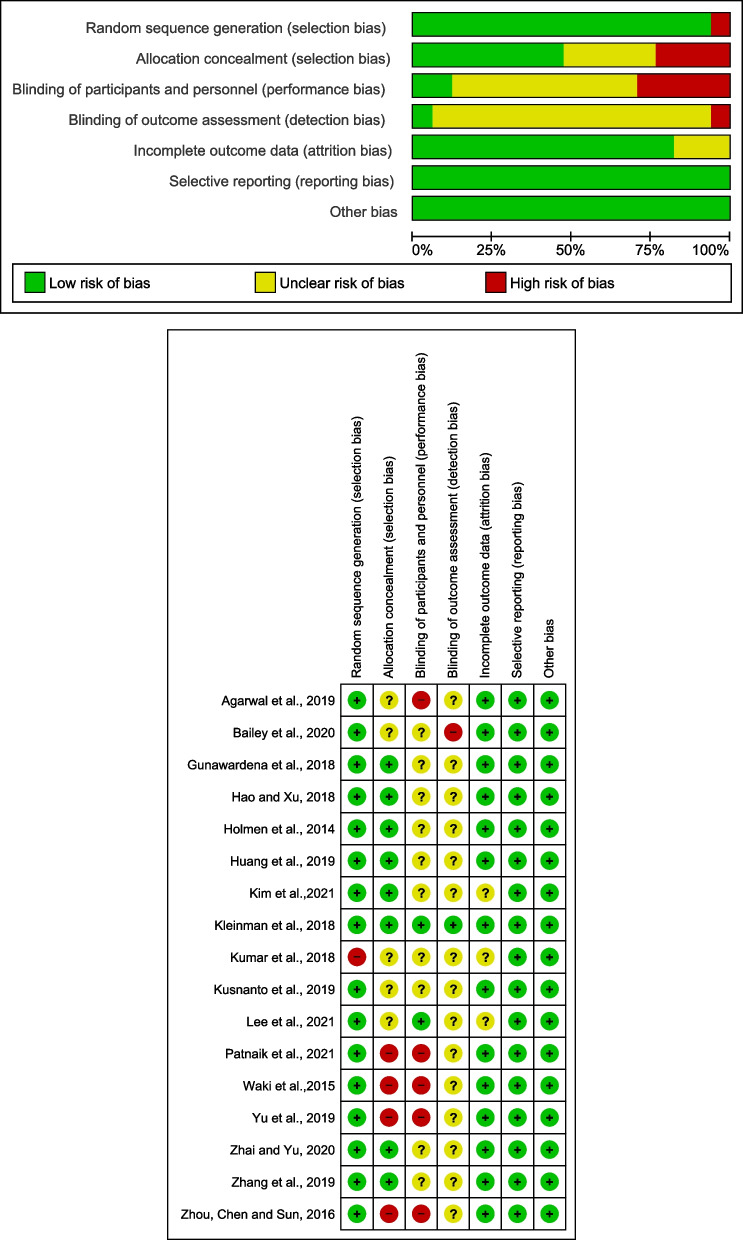
Summary risk of bias for each study included in the meta-analysis

We also carried out the specific risk of bias, which are shown in Fig. 4. In summary, 1 article fully met the quality criteria and 16 partially met these criteria, therefore, all were included in this study. Only 1 article did not report random sequence generation, presenting a high risk of bias. Most articles (14/17) presented results and follow-ups; therefore, the risk of attrition bias was low. With regard to reporting bias, all studies had this low risk of bias, as they clearly expressed the characteristics of participants in their respective studies.

### Quality assessment

Of the 26 articles included in the systematic review, 14 scored between 50 and 75% in the quality assessment, and the other studies presented criteria that classified them as being greater than 75% in the evaluation criteria of the Joanna Briggs Institute forms. No study was of low quality.

## Discussion

The results of our systematic review and meta-analyses confirm our hypothesis that the use of mobile health applications as a digital intervention improves the management, self-monitoring and monitoring of adult and elderly patients with AH and/or DM and, can be more effective than currently employed strategies. The meta-analysis showed that there is a difference between the intervention and control groups, indicating that the intervention group, that is, those who used mobile health applications, had a reduction of 0.39% (CI 0.24 – 0.54) in the HbA1c value (%) compared to the usual care group.

Other studies and analyzes support these findings, such as that by Sun et al. [19] with DM, Chinese and elderly patients who received glucometers that transmit data and received guidance on medication, diet and exercise through mHealth. After six months of intervention, patients exhibited a decreasing trend in HbA1c levels compared to baseline and the control group. Similar results of clinical improvement in this parameter were observed in other studies [20–23]. The study by Quinn et al. [24] and Kim et al. [25] showed that elderly patients with DM have good interaction in an educational environment developed within the mobile application with information and guidance about the disease, significantly improving self-monitoring and self-management of blood glucose levels. A similar result was obtained from the study by Mehraeen et al. [26], who concluded that blood sugar monitoring, exercise, nutrition, weight monitoring, and educational resources were the most outstanding technical features of the self-care app for type 2 diabetes.

Furthermore, we had a decrease in blood pressure levels and behavioral changes demonstrated in the studies included in the systematic review. Morawski et al. [27] were able to prove improvement in self-reported medication adherence and decrease in blood pressure levels. Bailey et al. [28] were also able to identify improvements in self-care, well-being, decrease in % body fat and glucose tolerance, with the use of an mHealth for eight weeks. A similar result was obtained in a study carried out for two years with the mobile application as an intervention, in which there was a significant improvement in glucose and diet control, improvement in physical activity and in general health care [29]. All these studies point to and corroborate what was concluded in the study by Mehraeen et al. [30], that the lack of self-care increases the likelihood of complications from the disease, therefore, focusing on self-care is one of the main strategies for changing behavior and lifestyle for this public.

The positive changes resulting from the intervention of digital health in the lives of patients affected by chronic diseases are undeniable and necessary. It is vital that healthcare professionals and patients come together to form an effective and integrative relationship in clinical practice with mobile health applications, as with the advancement of software and mobile technology, mHealth has become an important element in people's daily lives [31, 32]. The use of technology, especially apps as demonstrated in our systematic review and meta-analysis, is effective and considered successful for health care and assistance [33–35]. With regard to the treatment of chronic diseases and patient education on disease monitoring, it reinforces adherence to treatment, facilitating the understanding of recommendations and being easily accessible [36–38].

We also emphasize that of the 26 studies included in our systematic review and meta-analysis, 11 still approached the primary health care level or did not have information about the level of care where the study was developed. This demonstrates that many individuals who have chronic diseases such as DM and AH are not being treated at the first level of health care, where many risk factors for the emergence and negative progression of the disease are avoided. Still, studies carried out in primary care, specifically, demonstrated that the care, monitoring and management of these diseases through a mobile application helped in disease control and adherence to drug and non-drug treatment, as previously mentioned.

The present study presents important contributions to the area of digital health, which has grown significantly in recent years. Within the scope of our systematic review, we focused on the effectiveness of the use of mobile apps for health in the hypertensive and diabetic public, an audience that was left unattended during the COVID-19 pandemic and who are among the most vulnerable groups to develop complications arising from the contagion by the virus, in addition to being diseases that grow exponentially worldwide, which demonstrates the relevance of the study developed.

We have included five publicly accessible and comprehensive databases, which suggest the inclusion of relevant publications. Regarding the quality assessment, the quality of the articles included in the systematic review was high and it was possible to perform a meta-analysis with more than half of the articles included in the systematic review, without publication bias, with high overall strength of evidence of the effectiveness of the intervention studied and with randomized study with randomly selected sample, which demonstrates the high rigor of the analyzes performed. The average intervention time of the studies had minimal variation, with the majority being at least 3 months and a maximum of 12 months, which suggests good standardization and consistency of the included studies.

This study may have some limitations. The first is that most studies were carried out in Asia and Europe, not being a geographically comprehensive study. Second, some studies lacked double-blind selection. Finally, we were unable to perform a meta-analysis with blood pressure data due to the absence of these data at the beginning and end of the follow-up period and the respective standard deviation. The same happened with some important DM measurement parameters, such as fasting blood glucose, since this parameter was not very detailed in most of the studies included in our systematic review and meta-analysis. To reduce these limitations in future research, we suggest stricter inclusion criteria and that more parameters for measuring and monitoring DM and AH are inserted and analyzed more frequently, mainly at the primary care level.

## Conclusions

A total of 26 articles were included in the systematic review, of which 17 were eligible for meta-analysis. We focus on the effectiveness of using mobile applications for monitoring AH and/or DM. Our results showed that the use of mobile applications reduced HbA1c in 0.39% (CI 0.24 – 0.54), in addition to improving adherence to drug treatment and providing important behavioral changes, which include the practice of physical activity and weight reduction.

Thus, through a rigorous methodological approach, the findings of our systematic review and meta-analysis provided an overview of digital health, with a focus on mobile applications for the health of hypertensive and diabetic patients with a view to the ongoing digital transformation and the recommendations for best practices.

In summary, we noticed with our study that there is the use of mobile applications at different levels of care (primary, secondary and tertiary), all with success in clinical and behavioral parameters. However, for future research, we strongly recommend that research focus on identifying those users, especially among the elderly population, who have more difficulty using applications on a daily basis, as this difficulty exists and can be a limiting factor. Another perspective for the future is the incorporation of the different health systems within the mobile application, which will allow for a better relationship between professionals and patients.

### Supplementary Information

**Additional file 1.**

**Additional file 2.**

## Data Availability

All data included in this review and meta-analysis are reported in article/additional files.

## Abbreviations

AH: Arterial Hypertension
DM: Diabetes Mellitus
mHealth: Mobile health
PRISMA: Preferred Reporting Items for Systematic Reviews and MetaAnalyses
PROSPERO: International Prospective Register of Systematic Reviews
WMD: Weighted difference means
χ2: Chi-square test
I2: I-square
SD: Standard deviation
HbA1c: Glycated hemoglobin
CI: Confidence interval
