# Supplementary material for: The effectiveness of mobile application for monitoring diabetes mellitus and hypertension in the adult and elderly population: systematic review and meta-analysis
Source: BMC Health Serv Res. 2023 Aug 12;23:855. doi: 10.1186/s12913-023-09879-6 (PMC10423411; doi:10.1186/s12913-023-09879-6)
Supplement: Supplementary file 1 — Additional file 1. [file 12913_2023_9879_MOESM1_ESM.pdf]

Additional file 1

Chart 1. Study Characteristics

№	Title<br>Year of publication	Authors,<br>Country,<br>Year of study	Type of study	Disease and Level of health care	Sample size (I/C) *	Sample characterization	Health app name and purpose	Data of the studies and Outcomes	Quality of Studies**
1	The Effect of a Smartphone-Based, Patient-Centered Diabetes Care System in Patients With Type 2 Diabetes: A Randomized, Controlled Trial for 24 Weeks<br><br>2019	Kim, Y et al.<br><br>South Korea<br><br>2019	Randomized clinical trial	Type 2 Diabetes, tertiary health care	68 patients (32/36)	Individuals over 19 years old, with an HbA1C level greater than 6.5%, on drug treatment for more than 6 months.	mDiabetes<br><br>Eevaluated the efficacy of a smartphone-based, patient-centered diabetes care system (mDiabetes) for type 2 diabetes	In the experimental group, they improved with the decrease in HbA1c (Z=-4.83, p<0.001) in relation to the control group:<br>- Knowledge of diabetes self-management (Z = -2.70, p = 0.007),<br>- Social motivation (Z = -1.97, p = 0.048)<br>- The behavior (t = 3.22, p = 0.002)<br><br>HbA1c decreased 1.01±1.29% in the experimental group and 0.09±0.70% in the control group, statistically significant pre- and post-test differences between the two groups (Z=-4.83, p<, 001).	10/13
2	Impact of a mobile phone app on adherence to treatment regimens among hypertensive patients: A randomised clinical trial study<br><br>2021	Ibrahim Abu-El-Noor et al.<br><br>Gaza strip<br><br>2021	Randomized clinical trial	Hypertension, primary health care	191 patients (94/97)	The target population of the study were adults (over 18 years old) diagnosed with hypertension for at least one year prior to data collection and who had received a prescription for at least one antihypertensive medication.	Mobile phone app<br><br>Evaluate the impact of using a mobile phone app on the level of adherence to treatment regimens among hypertensive patients in the Gaza Strip.	The intervention group showed significant improvement compared to the control group in the following domains:<br>- Adherence to the use of the application (P = 0.000)<br>- Adherence to medication (P = 0.000)<br>- Adherence to the diet (P = 0.001)	10/13
3	Effect of a Smartphone App on Weight Change and Metabolic Outcomes in Asian Adults With Type 2 Diabetes<br><br>2021	Lim, SL et al.<br><br>Singapore<br><br>2017-2019	Randomized clinical trial	Type 2 Diabetes, primary and secondary health care	204 patients (105/99)	Individuals with physician-diagnosed T2DM, age between 21 and 75, body mass index (BMI) 23.0 or greater, English literacy, and access to a smartphone.	Nutritionist Buddy Diabetes<br><br>To compare the effects of a culturally contextualized smartphone-based intervention with usual care on weight and metabolic outcomes.	The intervention group showed significant improvement compared to the control group in the following domains:<br>- Body weight (mean [SD] weight change, -3.6 [4.7] kg vs -1.2 [3] .6] kg; P < 0.001)<br>- HbA1c levels: 0.7% (1.2) and 0.3% (1.0) in the intervention and control groups, respectively<br>- Fasting blood glucose: 14.4 (37.8) mg/ dL and 1.8 (25.2) mg/ dL in the intervention groups and control, respectively.<br>- Reduction of food consumption<br>- Increased levels of physical activity	10,5/13

4	Mobile health application based intervention for improvement of quality of life among newly diagnosed type 2 diabetes patients<br><br>2021	Patnaik, L et al.<br><br>India<br><br>2016-2018	Randomized clinical trial	Type 2 Diabetes, tertiary health care	66 patients (33/33)	Individuals with newly diagnosed T2DM, educated and technology-educated smartphone users, ages 18-60	Mobile health application<br><br>To know the usefulness of the mobile health application for improvement of QoL and diabetes self-management activities of the type 2 diabetes patients.	Overall quality of life scores was increased in both the control and intervention groups during follow-ups. It was found that there was significant improvement in glucose management, dietary control, physical activity, health care and sum score. Wilk's lambda was significant for HbA1c in both the control and intervention groups	10,5/13
5	Effects of Short-term Mobile Application Use on Weight Reduction for Patients with Type 2 Diabetes<br><br>2021	Lee, S.E et al.<br><br>South Korea<br><br>2019-2020	Randomized clinical trial	Type 2 Diabetes, and Hypertension, secondary health care	50 patients (25/25)	Individuals with T2DM with a glycosylated hemoglobin (HbA1c) level ≥6.5% who were being treated with antidiabetic medications.	LIBIT application<br><br>Investigate the effects of a mobile application on weight reduction in patients with T2DM.	Patients in both groups experienced similar changes in mean body weight (from 68.3 kg to 69.7 kg in the control group and from 71.4 kg to 72.0 kg in the intervention group), with no difference between groups (P = 0.229). Regarding changes in WC, the control group showed an increase in WC (from 95.00±8.89 cm to 95.76±9.72 cm), while the intervention group showed a reduction (from 91.93±6.25 cm to 90.75±6.01 cm) (P = 0.016).	10/13
6	A Mobile Health Intervention for Self-Management and Lifestyle Change for Persons With Type 2 Diabetes, Part 2: One-Year Results From the Norwegian Randomized Controlled Trial RENEWING HEALTH<br><br>2014	Holmen et al.<br><br>Norway<br><br>2014	Randomized clinical trial	Type 2 Diabetes, primary health care	151 patients (50/101)	T2DM patients aged over 18 years, who had an HbA1 level≥7.1% (54.1 mmol/mol), and were able to complete questionnaires in the Norwegian language.	Few Touch Application (FTA)<br><br>The objective of this study was to test whether the use of a mobile phone–based self-management system used for 1 year, could improve glycated hemoglobin A1c (HbA1c) level, self-management, and health-related quality of life compared with usual care.	The change in HbA1c level did not differ significantly between the 3 groups after 1 year. However, HbA1c dropped within all groups.	10/13
7	DialBetics With a Multimedia Food Recording Tool, FoodLog: Smartphone-Based Self-Management for Type 2 Diabetes<br><br>2015	Waki, K et al.<br><br>Japan<br><br>2015	Clinical trial	Type 2 Diabetes, N/A	59 patients (32/27)	In the 3-month study, patients with T2DM. In the 1-week study, 5 patients with T2DM without serious complications and diagnosed more than 2 years ago, who had the ability to exercise.	DialBetics<br><br>Test a more patient-friendly version of DialBetics, whose development was based on the original participants' feedback about the previous version of DialBetics.	HbA1c had declined a significant 0.4% among those who used DialBetics compared with the control group.	9/13
8	Welltang – A smart phone-based diabetes management application – Improves blood glucose control in Chinese people with diabetes<br><br>2016	Zhou et al.<br><br>China<br><br>2016	Randomized clinical trial	Type 1 and 2 Diabetes and Hypertension, N/A	100 patients (50/50)	Individuals aged between 18 and 74, diabetic, without serious complications and able to use a smartphone	Welltang<br><br>To evaluate the impact of the smart phone-based diabetes management application, Welltang, on glycated hemoglobin (HbA1c).	Mean decrease in HbA1c was 1.95% (21 mmol/mol) in the intervention group and 0.79% (8 mmol/mol) in the control group (P < 0.001). Self- monitored blood glucose measurements, diabetes knowledge, and self-care behaviors improved in patients in the intervention group. 84% of patients in the intervention group were satisfied with the use of the application.	8,5/13
9	Improved Medication Adherence and Frequency of Blood Glucose Self-Testing using an m-Health Platform Versus Usual Care in a	Kleinman, et al.<br><br>India<br><br>2017	Randomized clinical trial	Type 2 Diabetes and Hypertension, N/A	90 patients (39/41)	Men or non-pregnant women, aged between 18 and 65 years, with T2DM for at least 6 months, who do not use an insulin pump,	m-Health intervention<br><br>The impact of an m-Health diabetes platform on clinical outcomes, patient-reported	Intervention group patients had a mean HbA1c decrease of 1.5%, while the control group of 0.8% (p = 0.020).	10,5/13

	Multisite Randomized Clinical Trial Among People with Type 2 Diabetes in India<br><br>2017					glucocorticoids or continuous glucose monitor.	outcomes, patient and provider satisfaction, and app usage.		
10	Association of a Smartphone Application With Medication Adherence and Blood Pressure Control<br><br>2018	Morawski et al.<br><br>EUA<br><br>2018	Randomized clinical trial	Hypertension, N/A	411 patients (202/209)	Individuals aged 18 to 75 years with a systolic blood pressure of 140 mm Hg or greater receiving treatment with at least 1 but not more than 3 first-line antihypertensive drugs	Medisafe smartphone app<br><br>To determine if the Medisafe smartphone app improves self-reported medication adherence and blood pressure control.	Mean medication adherence in the intervention group increased by 0.4 (1.5), while in the control group it remained unchanged. Regarding blood pressure, mean systolic blood pressure decreased (10.6 - 16.0 mmHg) in the intervention group and in the control group (10.1 -15.4 mmHg). There was no significant difference in blood pressure change between groups.	10/13
11	A Prospective Cohort study on the Management of Young Patients with Newly Diagnosed Type 2 Diabetes Using Mobile Medical Applications<br><br>2018	Hao, Y.; Xu, H.;<br><br>China<br><br>2018	Prospective cohort	Type 2 Diabetes, N/A	126 patients (66/60)	Individuals aged between 18 and 40 years, non-pregnant, diagnosed with T2DM.	Mobile medical app<br><br>Compare the data obtained by the mobile application with the data obtained from patients receiving traditional medicine treatment.	At 24 weeks of treatment, HbA1c, triglyceride and LDL-C levels in both groups were significantly lower than their respective baseline values.<br>HbA1c was significantly higher in the intervention group than in the control group.	8/11
12	Digital Diabetes Management Application Improves Glycemic Outcomes in People With Type 1 and Type 2 Diabetes<br><br>2017	Offringa et al.<br><br>China<br><br>2018	retrospective cohort	Type 1 and 2 Diabetes, N/A	1799 patients (900/899)	Individuals with DM1 OR T2DM, who had at least 90 days (i.e., approximately 3 months) of data prior to their initial upload to the app.	Mobile health management software<br><br>Determine the real-world glycemic benefits of a mobile diabetes management platform used by individuals with type 1 and type 2 diabetes.	The intervention group had fewer hyperglycemic events and higher mean glucose levels compared to the control group. In addition, patients in the intervention group had a 3.5% decrease in mean blood glucose (-6.4 mg/dL, 95% CI [-2.0, -10.7], P <.001) and a decrease in of 10.7% in hyperglycemia (P<.001) after 2 months.	8,5/11
13	A Diabetes Mobile App With In-App Coaching From a Certified Diabetes Educator Reduces A1C for Individuals With Type 2 Diabetes<br><br>2018	Kumar et al.<br><br>EUA<br><br>2018	Prospective cohort	Type 2 Diabetes, N/A	146 patients (126/20)	Individuals aged 18-75 with self-reported T2DM and A1C≥ 7.5% (58 mmol/mol) who indicated they were motivated to use a daily diabetes self-management training program.	One Drop/Mobile With One Drop/Experts<br><br>To evaluate the impact of a diabetes mobile app with in-app coaching by a certified diabetes educator on glycemic control for individuals with type 2 diabetes.	Mean of A1C improved by −0.86% among study completers (n = 127), −0.96% among active users of the app and coaching program (n = 93), and −1.32% among active users with a baseline A1C ≥9.0% (75 mmol/mol) (n = 53).	7/11
14	The Influence of the Smart Glucose Manager Mobile Application on Diabetes Management<br><br>2018	Kasun C Gunawardena et al.<br><br>Sri Lanka<br><br>2018	Randomized clinical trial	Diabetes Mellitus, primary health care	67 patients (27/25)	Patients between 18 and 80 years old diagnosed with T2DM at least 6 months before the survey, HbA1c above 8.0%.	Smart Glucose Manager (SGM)<br><br>Investigated the efficacy of a newly designed mobile application, Smart Glucose Manager (SGM), in Sri Lankan patients with diabetes.	Long-term improvement in HbA1c levels.	9,5/13
15	Effects of mobile phone application combined with or without self-monitoring of blood glucose on glycemic control in patients with diabetes: A randomized controlled trial	Yuan Yu et al.<br><br>China<br><br>2019	Randomized clinical trial	Diabetes Mellitus, primary health care	185 patients (47/138)	Men and women aged 35 to 65 years, with T2DM, able to use a cell phone, with no HbA1c limit.	Mobile phone application (MPA)<br><br>Evaluate the effects of a mobile phone application (MPA) combined with or	Improves HbA1c ratio <7% in patients with type 2 diabetes.	10/13

	2019						without self-monitoring of blood glucose (SMBG) on glycemic control in patients with diabetes.		
16	Specific hypertension smartphone app to improve medication adherence in hypertension: a cluster-randomized trial<br><br>2018	Marquez Contreras, et al.<br><br>Spain<br><br>2016-2017	Randomized clinical trial	Hypertension, secondary health care	154 patients (77/77)	Patients over 18 years of age diagnosed with hypertension on drug treatment for at least one month.	Mobile phone app<br><br>To evaluate the effectiveness of the intervention in pharmacological therapeutic adherence of the mild-moderate arterial hypertension (AHT), through an APP installed in the mobile phone.	The application favors pharmacological therapeutic adherence, improves blood pressure, improves adherence.	9,5/13
17	DM-calendar app as a diabetes self-management education on adult type 2 diabetes mellitus: a randomized controlled trial<br><br>2019	Kusnanto, et al.<br><br>Indonesia<br><br>2018	Randomized clinical trial	Type 2 Diabetes, primary health care	65 patients (30/35)	Patients diagnosed with T2DM in the last year, capable of operating an android cell phone, with an uncontrolled glycemic profile.	Diabetes mellitus calendar app<br><br>The aim was to evaluate the effect of diabetes mellitus calendar app as a Diabetes Self-Management Education (DSME) program on self-efficacy, HbA1c levels, lipid profile, and insulin in adult type 2 diabetes mellitus (T2DM).	Increases self-efficacy, decreases HbA1c, improves lipid profile and insulin levels in patients with T2DM.	9/13
18	Integrated Diabetes Self-Management Program Using Smartphone Application: A Randomized Controlled Trial<br><br>2021	Kim, EK et al.<br><br>South Korea<br><br>2017-2018	Randomized clinical trial	Type 2 Diabetes and Hypertension, secondary health care	151 patients (94/97)	Patients aged 19 to 80 years diagnosed with T2DM in oral treatment for at least 3 months with Hb1Ac levels between 7.0 and 10%.	Information-Motivation-Behavioral skills (IMB) model.<br><br>To develop an IMB model-based integrated self-management program that could improve self-management behavior for type 2 diabetes patients through a diabetes self-management smartphone app.	Significant reduction in HbA1c levels, with tolerable safety profiles.	9/13
19	Mobile App for Improved Self-Management of Type 2 Diabetes: Multicenter Pragmatic Randomized Controlled Trial<br><br>2019	Agarwal et al.<br><br>Canada<br><br>2018	Randomized clinical trial	Type 2 Diabetes, primary health care	223 patients (113/110)	Patients over 18 years old, diagnosed with T2DM, Hb1Ac > 8.0%.	BlueStar mobile app<br><br>To determine if app usage leads to improved HbA1c levels among diverse participants in real-life clinical contexts	No significant differences were found in the dosage of Hb1Ac in intervention and control groups	11/13
20	A Smartphone App to Improve Medication Adherence in Patients With Type 2 Diabetes in Asia: Feasibility Randomized Controlled Trial<br><br>2019	Huang et al.<br><br>Singapore<br><br>2018	Randomized clinical trial	Type 2 Diabetes, primary health care	41 patients (26/25)	Patients between 21 and 75 years old with T2DM and the ability to use a cell phone.	Medisafe app<br><br>Determine the feasibility, acceptability, and clinical outcomes of using a smartphone app to improve medication adherence in a multiethnic Asian population with T2DM.	It improved knowledge about the disease, medication adherence, but did not improve clinical outcomes.	9,5/13
21	Effectiveness of Smartphone App–Based Interactive Management on Glycemic Control in Chinese Patients With Poorly Controlled	Zhang et al.<br><br>China<br><br>2015-2016	Randomized clinical trial	Type 2 Diabetes and Hypertension, primary health care	276 patients (92/184)	Patients aged 18 to 65 years with DM1 or T2DM diagnosed for 3 months and Hb1Ac >8.0%.	App self-management<br><br>Investigate the effectiveness of an app-based interactive management model by	The effect of self-management alone in patients in the intervention group was not superior to that of routine management.	9/13

	Diabetes: Randomized Controlled Trial<br><br>2019						a professional health care team on glycemic control in Chinese patients with poorly controlled diabetes.		
22	Randomised Controlled Feasibility Study of the MyHealthAvatar-Diabetes Smartphone App for Reducing Prolonged Sitting Time in Type 2 Diabetes Mellitus<br><br>2020	Bailey et al.<br><br>United Kingdom<br><br>2020	Randomized clinical trial	Type 2 Diabetes and Hypertension, secondary health care	20 patients (10/10)	Men and women between 18 and 65 years of age T2DM diagnosed in the last 4 years.	MyHealthAvatar-Diabetes<br><br>To evaluate the feasibility and acceptability of a self-regulation smartphone app with targeted BCTs for reducing prolonged sitting in people with T2DM.	There were preliminary improvements in the number of breaks in sitting per day, body fat %, glucose tolerance, attitude, intention, planning, wellbeing, and positive and negative affect in favour of the intervention group.	9/13
23	The impact of a pharmacist-designed mobile application on blood pressure control and medication adherence in patients with hypertension<br><br>2020	Manigault et al.<br><br>EUA<br><br>2016-2018	Randomized clinical trial	Hypertension, primary health care	78 patients (39/39)	Patients over 18 years of age with hypertension using a medication for 3 months.	Mobile applications<br><br>To determine the effectiveness of using a pharmacist-designed app to improve blood pressure (BP) and promote adherence to antihypertensive medications.	The intervention group did not result in better medication adherence or blood pressure control.	8/13
24	Impact of a comprehensive digital health programme on HbA1c and weight after 12 months for people with diabetes and prediabetes: a randomised controlled trial<br><br>2020	McLeod et al.<br><br>Germany<br><br>2017-2018	Randomized clinical trial	Diabetes Mellitus and Hypertension, primary health care	429 patients (214/215)	Individuals aged 18-75 years, HbA1C –70 mmol/mol (5.9–8.6%), who do not take insulin and have daily internet access.	BetaMe/Melon<br><br>To evaluate the effectiveness of a digital health programme (BetaMe/Melon) vs usual care in improving the control of T2DM and prediabetes in a primary care population.	This study did not demonstrate efficacy clinic for this specific program.	11,5/13
25	My Diabetes Coach, a Mobile App–Based Interactive Conversational Agent to Support Type 2 Diabetes Self-Management: Randomized Effectiveness-Implementation Trial<br><br>2020	Gong et al.<br><br>Australia<br><br>2016-2017.	Randomized clinical trial	Type 2 Diabetes and Hypertension, primary health care	187 patients (94/93)	Adults over 18 years of age diagnosed with T2DM in the last 10 years.	My Diabetes Coach (MDC)<br><br>To evaluate the adoption, use, and effectiveness of the My Diabetes Coach (MDC) to support diabetes self-management in the home setting over 12 months.	The program has been successfully adopted by individuals with T2DM, improving their quality of life.	11/13
26	A Mobile App for diabetes Management: Impact on Self-Efficacy Among Patients with Type 2 Diabetes at a Community Hospital<br><br>2020	Yangkui Zhai, Wenjuan Yu<br><br>China<br><br>2018-2019	Prospective randomized trial	Type 2 Diabetes, secondary health care	120 patients (60/60)	Patients diagnosed with T2DM for more than 3 months, between 18 and 60 years old who know how to use a cell phone and do not intend to travel in the next 6 months.	One Drop/Mobile With One Drop/Experts<br><br>The impact of a diabetes mobile app and coaching program.	The diabetes app intervention helped patients with T2DM improve clinical care and self-management behavior.	9,5/13

*I= intervention group, C=control group.
** The score refers to the type of study of each article. For the type of study, there is a specific instrument by Joanna Briggs, which varies the total number of questions.
N/A: not applicable; HbA1C: glycated hemoglobin; T2DM: tipe 2 diabetes.
